# Multilevel analyses of related public health indicators: The European Surveillance of Congenital Anomalies (EUROCAT) Public Health Indicators

**DOI:** 10.1111/ppe.12655

**Published:** 2020-02-26

**Authors:** Kate E. Best, Judith Rankin, Helen Dolk, Maria Loane, Martin Haeusler, Vera Nelen, Christine Verellen‐Dumoulin, Ester Garne, Gerardine Sayers, Carmel Mullaney, Mary T. O'Mahony, Miriam Gatt, Hermien De Walle, Kari Klungsoyr, Olatz Mokoroa Carolla, Clara Cavero‐Carbonell, Jennifer J. Kurinczuk, Elizabeth S. Draper, David Tucker, Diana Wellesley, Nataliia Zymak‐Zakutnia, Nathalie Lelong, Babak Khoshnood

**Affiliations:** ^1^ Institute of Health & Society Newcastle University Newcastle upon Tyne UK; ^2^ Centre for Maternal, Fetal and Infant Research Institute of Nursing and Health Research Ulster University Ulster UK; ^3^ Medical University of Graz Graz Austria; ^4^ Provinciaal Instituut voor Hygiëne Antwerp Belgium; ^5^ Eurocat Hainaut –Namur Centre for Human Genetics Institut de Pathologie et de Génétique, IPG Charleroi Belgium; ^6^ Paediatric Department Hospital Lillebaelt Kolding Denmark; ^7^ Health Intelligence Health Service Executive Dublin Ireland; ^8^ Public Health Department HSE Southeast area Lacken Kilkenny Ireland; ^9^ Department of Public Health Health Service Executive South Cork Ireland; ^10^ Department of Health Information and Research Guardamangia Malta; ^11^ Department of Genetics University Medical Center Groningen University of Groningen Groningen The Netherlands; ^12^ Department of Global Public Health and Primary Care University of Bergen Bergen Norway; ^13^ Public Health Division of Gipuzkoa BioDonostia Research Institute San Sebastian Spain; ^14^ Rare Diseases Research Unit Foundation for the Promotion of Health and Biomedical Research of the Valencian Region Valencia Spain; ^15^ National Perinatal Epidemiology Unit Nuffield Department of Population Health University of Oxford Oxford UK; ^16^ Department Health Sciences University of Leicester Leicester UK; ^17^ Congenital Anomaly Register and Information Service for Wales Public Health Wales Swansea UK; ^18^ Faculty of Medicine University of Southampton and Wessex Clinical Genetics Service Southampton UK; ^19^ OMNI‐Net Ukraine and Khmelnytsky Children Hospital Khmelnytsky Ukraine; ^20^ INSERM U1153 (Obstetrical, Perinatal and Pediatric Epidemiology Research Team, Center for Biostatistics and Epidemiology) Maternité Port Royal Paris France

**Keywords:** perinatal mortality, termination of pregnancy for foetal anomaly

## Abstract

**Background:**

Public health organisations use public health indicators to guide health policy. Joint analysis of multiple public health indicators can provide a more comprehensive understanding of what they are intended to evaluate.

**Objective:**

To analyse variaitons in the prevalence of congenital anomaly‐related perinatal mortality attributable to termination of pregnancy for foetal anomaly (TOPFA) and prenatal diagnosis of congenital anomaly prevalence.

**Methods:**

We included 55 363 cases of congenital anomalies notified to 18 EUROCAT registers in 10 countries during 2008‐12. Incidence rate ratios (IRR) representing the risk of congenital anomaly‐related perinatal mortality according to TOPFA and prenatal diagnosis prevalence were estimated using multilevel Poisson regression with country as a random effect. Between‐country variation in congenital anomaly‐related perinatal mortality was measured using random effects and compared between the null and adjusted models to estimate the percentage of variation in congenital anomaly‐related perinatal mortality accounted for by TOPFA and prenatal diagnosis.

**Results:**

The risk of congenital anomaly‐related perinatal mortality decreased as TOPFA and prenatal diagnosis prevalence increased (IRR 0.79, 95% confidence interval [CI] 0.72, 0.86; and IRR 0.88, 95% CI 0.79, 0.97). Modelling TOPFA and prenatal diagnosis together, the association between congenital anomaly‐related perinatal mortality and TOPFA prevalence became stronger (RR 0.70, 95% CI 0.61, 0.81). The prevalence of TOPFA and prenatal diagnosis accounted for 75.5% and 37.7% of the between‐country variation in perinatal mortality, respectively.

**Conclusion:**

We demonstrated an approach for analysing inter‐linked public health indicators. In this example, as TOPFA and prenatal diagnosis of congenital anomaly prevalence decreased, the risk of congenital anomaly‐related perinatal mortality increased. Much of the between‐country variation in congenital anomaly‐related perinatal mortality was accounted for by TOPFA, with a smaller proportion accounted for by prenatal diagnosis.

**Synopsis**

**Study question**

How can we analyse inter‐linked public health indicators?

**What's already known**

Public health indicators are commonly used to inform policy decisions. Public health indicators in the same area are often linked, meaning variation in one indicator may be associated with variations in another. Nevertheless, public health indicators are usually considered separately.

**What this study adds**

Using multilevel analysis, we present a novel method for modelling the association between public health indicators that takes into account the hierarchical nature of data used to measure health indicators. Specifically, the method can be used to explore how, and the extent to which, different public health indicators may be related to one another.

## 1 INTRODUCTION

Public health indicators are commonly used to assess a population's health status. They are intended as an effective and uncomplicated way to compare public health between populations and over time.^1^, ^2^ Public health organisations, including the World Health Organisation (WHO) and the European Commission, use public health indicators to evaluate interventions and guide policy.^3^, ^4^

Congenital anomalies are an important public health concern given that they are major contributors to infant mortality, morbidity in childhood and lifelong disability.^5^ The European Surveillance of Congenital Anomalies (EUROCAT) is a collaborative network of 43 population‐based congenital anomaly registers, based in 23 countries.^6^ The registries collect data on congenital anomalies occurring in live births, late miscarriages (20‐24 weeks gestation), stillbirths (>24 weeks gestation), and termination of pregnancy for foetal anomaly (TOPFAs, any gestation). The registries each use multiple sources to ascertain cases and their outcomes, including hospital records (antenatal ultrasound, foetal medicine, cytogenetic laboratories, and paediatric surgery), birth and death certificates, and post‐mortem examinations.^7^ Registries vary in the age up to which they ascertain new diagnoses, but most registries are notified of cases diagnosed up to at least age 1 year.^7^ EUROCAT surveys approximately 1.7 million births per year in Europe, representing 29% of the European birth population.^6^

EUROCAT have developed six public health indicators that aim to evaluate the public health impact and assessment of interventions for congenital anomalies in Europe, using data routinely recorded by each registry.^8^ These indicators, include: (a) the prevalence of congenital anomaly‐related perinatal mortality per 1000 total births; (b) the prevalence of prenatal diagnosis of congenital anomaly per 1000 total births; (c) the prevalence of TOPFA following prenatal diagnosis of a congenital anomaly per 1000 total births; (d) Down's syndrome live birth prevalence per 1000 total births; (e) the prevalence of congenital anomalies requiring paediatric surgery per 1000 total births; and (f) the prevalence of neural tube defects per 1000 total births. Previous examination of these indicators suggested that countries with very low TOPFA rates had higher prevalence of congenital anomaly‐related perinatal mortality, although this was not formally tested.^8^

Outside of the perinatal setting, previous studies have examined the association between several public health indicators relating to a range of conditions.^1^, ^9^, ^10^ However, we are not aware of any studies, in the perinatal setting or otherwise, that have quantified the degree of variation in one indicator that may be attributable to another using variance explained measures in multilevel models. This approach may better explain relationships between inter‐linked public health indicators, in all areas of research.

The aim of this study was to demonstrate a multilevel statistical method of analysing inter‐linked public health indicators using an interesting example of EUROCAT public health indicators. Using our proposed method, we will show how to quantify what proportion of between‐country variation in congenital anomaly‐related perinatal mortality may be accounted for by TOPFA prevalence. We will also test the hypothesis that increased TOPFA prevalence is associated with decreased congenital anomaly‐related perinatal mortality rates.

## 2 METHODS

### 2.1 Data

Data on congenital anomalies notified to 18 full member EUROCAT registers in 10 countries between 1 January 2008 and 31 December 2012 were included in this study (Table 1). In order to restrict our analysis to countries with high ascertainment of congenital anomaly‐related perinatal mortality, we used Boyle et als' approach of excluding countries where the EUROCAT infant mortality rate (in cases of congenital anomaly) was more than 20% lower than that reported by the WHO between 2005 and 2009.^11^ EUROCAT defines a case of congenital anomaly as any case with at least one diagnosis in the Q chapter of the WHO International Classification of Disease (ICD) version 10, along with a limited set of conditions coded outside the Q chapter: D215, D821, D1810, P350, P352, and P371. Minor anomalies (eg, skin tags) are excluded if they occur in isolation; further details are available in the EUROCAT Guide 1.4.^12^ There were 55 363 cases with congenital anomalies notified to the 18 participating registers, which covered 2 430 440 total births during the study period. Indicators for the 18 registers were pooled according to country.

**Table 1:** Prevalence of EUROCAT public health indicators, by country

Country (Registers)	Total births	Perinatal mortality		TOPFA		Prenatal diagnosis	
		N	Prevalence per 1000 total births (95% CI)	N	Prevalence per 1000 total births (95% CI)	N	Prevalence per 1000 total births (95% CI)
Austria (Styria)	51 569	42	0.8 (0.6, 1.1)	197	3.8 (3.3, 4.4)	507	9.8 (9.0, 10.7)
Belgium (Antwerp, Hainaut)	170 449	153	0.9 (0.8, 1.1)	675	4.0 (3.7, 4.3)	707^a^	10.9 (10.1, 11.8)^a^
Denmark (Odense)	25 109	16	0.6 (0.4, 1.043)	172	6.9 (5.9, 8.0)	300	12.0 (10.6, 13.4)
Ireland (Dublin, SE Ireland, Cork and Kerry)	227 889	492	2.2 (2.0, 2.4)	38	0.2 (0.1, 0.2)	420^b^	4.6 (4.2, 5.1)^b^
Malta	21 013	66	3.1 (2.4, 4.0)	0	0	97	4.6 (3.7, 5.6)
Netherlands (N Netherlands)	87 415	91	1.0 (0.8, 1.3)	380	4.4 (3.9, 4.8)	852	9.8 (9.1, 10.4)
Norway	310 634	230	0.7 (0.7, 0.8)	1186	3.8 (3.6, 4.0)	2006	6.5 (6.2, 6.8)
Spain (Basque Country, Valencia Region)	344 576	165	0.5 (0.4, 0.6)	1802	5.2 (5.0, 5.5)	2894	8.4 (8.1, 8.7)
UK (Northern England, Thames Valley, East Midlands & South Yorkshire, Wales, Wessex)	1 033 724	1127	1.1 (1.0, 1.2)	5405	5.2 (5.1, 5.4)	12 252	11.9 (11.6, 12.1)
Ukraine	158 062	240	1.5 (1.3, 1.7)	539	3.4 (3.1, 3.7)	1230	7.8 (7.4, 8.2)

a Prenatal diagnosis available for Hainaut only.

b Prenatal diagnosis available for Cork and Kerry and SE Ireland but not Dublin.

### 2.2 Indicator definitions

The following indicators were used for each included country:

1. The prevalence of congenital anomaly‐related perinatal mortality per 1000 total births: calculated as the number of cases with one or more congenital anomalies that resulted in foetal death ≥20 weeks gestational age or a death within the first week after birth, divided by the total number of live births and stillbirths in the country of interest, multiplied by 1000.
2. The prevalence of termination of pregnancy for foetal anomaly (TOPFA) per 1000 total births*:* calculated as the number of terminations of a pregnancy (at any gestation) following prenatal diagnosis of a congenital anomaly, divided by the total number of live births and stillbirths in the country of interest, multiplied by 1000.
3. The prevalence of prenatal diagnosis of a congenital anomaly per 1000 total births: defined as the number of prenatally diagnosed cases with a congenital anomaly divided by the total number of live births and stillbirths in the country of interest, multiplied by 1000.

### 2.3 Statistical analysis

Prevalence rates for each indicator were calculated by country, with the corresponding 95% Poisson confidence intervals (CI). A null multilevel Poisson model (ie, a model with no explanatory variables) was fitted with congenital anomaly‐related perinatal mortality prevalence as the outcome (or rather, with congenital anomaly‐related perinatal mortality counts as the outcome and the log of the number of population births divided by 1000 as the offset) and a random effect for country, represented by the index *j* (Model 0):

$$\ln\left(\lambda \right)=\beta_{0j},$$

$$\beta_{0j}=\gamma_{00}+\mu_{0j}\;\text{variance}\;\left(\mu_{0j}\right)=\tau_{00},$$

where *λ* is the congenital anomaly‐related perinatal mortality indicator that varies across countries, as represented by the index *j*. The variance term *τ*_00_ represents “baseline” variations in the prevalence of perinatal mortality between countries.

In Model 1, the univariable association between (continuous) TOPFA prevalence and congenital anomaly‐related perinatal mortality prevalence was modelled:

$$\beta_{0j}=\gamma_{00}+\gamma_{01}\text{TOPFA}+\mu_{0j}\;\text{variance}\;\left(\mu_{0j}\right)=\tau_{00}^{*}.$$

In model 2, the univariable association between (continuous) prenatal diagnosis of congenital anomaly prevalence and congenital anomaly‐related perinatal mortality prevalence is modelled:

$$\beta_{0j}=\gamma_{00}+\gamma_{01}\text{Prenatal diagnosis}+\mu_{0j}\;\text{variance}\;\left(\mu_{0j}\right)=\tau_{00}^{\psi }.$$

It is also possible to fit a multivariable model, in order to control for additional variables. To demonstrate, Model 3 was fitted with both our explanatory variables included:

$$\beta_{0j}=\gamma_{00}+\gamma_{01}\text{TOPFA}+\gamma_{02}\text{Prenatal diagnosis}+\mu_{0j}\;\text{variance}\;\left(\mu_{0j}\right)=\tau_{00}^{*\psi }.$$

We can now use the models to give us two different types of information. Firstly, *γ*_01_ and *γ*_02_ from the fixed effects part of the model can be interpreted as incidence rate ratios (IRRs), for example *γ*_01_ in model 1 can be interpreted as the relative risk of congenital anomaly‐related perinatal mortality per unit increase in TOPFA prevalence. These show how the outcome and explanatory indicators are associated, for example the IRRs in models 1 and 2 can test the hypothesis that the risk of congenital anomaly‐related perinatal mortality decreases as TOPFA and prenatal diagnosis of congenital anomaly prevalence increase. In summary, the fixed effects can be used to test hypotheses about the association (linear or otherwise) between the outcome indicator and the explanatory indicators.

Secondly, the degree of variation in the outcome indicator that is “explained” by the explanatory indicator(s) can be calculated from the random effects part of the model. The variation between countries in the null model, which we refer to as the “baseline” variation, can be compared with the residual variation that remains after adjusting for explanatory indicators. This value can then be expressed as a percentage of the “baseline” variation between countries. So in our case, we can subtract the between‐country variation after taking into account TOPFA and prenatal diagnosis of congenital anomaly prevalence ($\tau_{00}^{*\psi }$) from the baseline between‐country variance (*τ*_00_). The difference between the baseline and residual variance is then divided by the baseline variance in order to calculate a percentage of the total variance:

$$\frac{\tau_{00}-\tau_{00}^{*\psi }}{\tau_{00}}\times 100.$$

In this example, this statistic can be interpreted as the percentage of between‐country variation in congenital anomaly‐related perinatal mortality prevalence accounted for by TOPFA and prenatal diagnosis of congenital anomaly prevalence.

Analyses were performed in Stata 14 (using the mepoisson command).

### 2.4 Missing data

Prenatal diagnosis data were not available for Antwerp at the time of analysis, and therefore, the prenatal diagnosis rate for Hainaut was used for Belgium. Prenatal diagnosis was not available for Dublin, and therefore, prenatal diagnosis prevalence for Ireland relates to Cork and Kerry and SE Ireland only.

### 2.5 Ethics approval

Data were downloaded in aggregate form, and therefore ethical approval was not required for this study. However, each EUROCAT register has their own ethical approvals in place for the collection of data without consent.

## 3 RESULTS

The prevalence of perinatal mortality in cases of congenital anomalies ranged from 0.5 (95% CI 0.4, 0.6) per 1000 total births in Spain to 3.1 (95% CI 2.4, 4.0) per 1000 total births in Malta (Table 1). The prevalence of TOPFA was lowest in Malta (0 per 1000 total births) where TOPFA is illegal, and greatest in Denmark (6.9 per 1000 total births, 95% CI 5.9, 8.0) (Table 1). The prevalence of prenatal diagnosis of congenital anomaly ranged from 4.6 per 1000 births in both Malta (95% CI 3.7, 5.6) and Ireland (95% CI 4.2, 5.1) to 11.95 (95% CI 10.6, 13.4) per 1000 total births in Denmark.

### 3.1 Fixed effect associations

**Figure 1:**
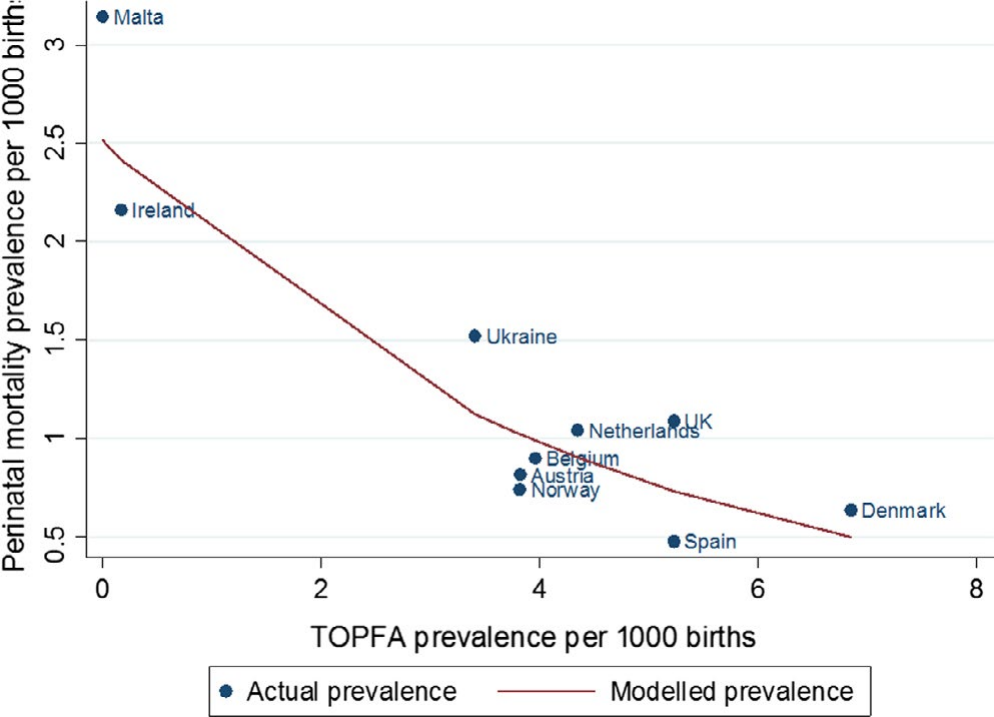
Actual and modelled association between perinatal mortality and TOPFA prevalence

**Figure 2:**
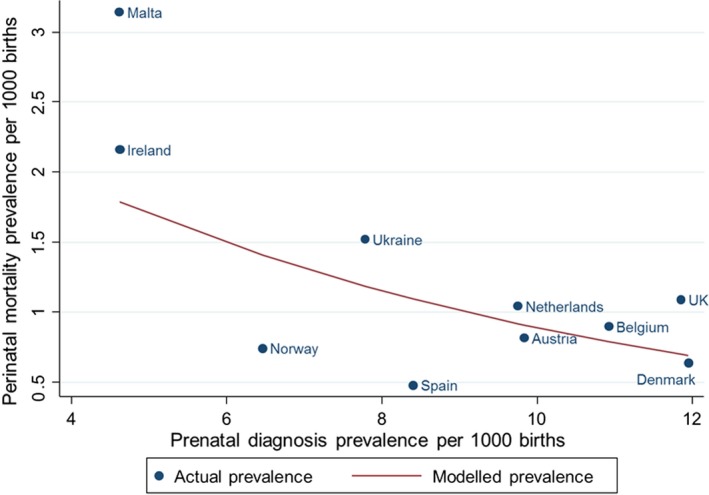
Actual and modelled association between perinatal mortality and prenatal diagnosis prevalence. ^¥^Prenatal diagnosis available for Hainaut only, ^†^Prenatal diagnosis available for Cork and Kerry and SE Ireland but not Dublin

There was a univariable linear association between TOPFA prevalence and congenital anomaly‐related perinatal mortality prevalence, with the risk of congenital anomaly‐related perinatal mortality decreasing as TOPFA prevalence increased (Model 1, IRR 0.79, 95% CI 0.72, 0.86) (Figure 1). There was also a univariable linear association between prenatal diagnosis of congenital anomaly and congenital anomaly‐related perinatal mortality prevalence, with the risk of perinatal mortality decreasing with increasing prenatal diagnosis prevalence (Model 2, IRR 0.88, 95% CI 0.79, 0.97; Figure 2). In model 3, the association between congenital anomaly‐related perinatal mortality prevalence and TOPFA prevalence strengthened after controlling for prenatal diagnosis prevalence (model 3, IRR 0.70, 95% CI 0.61, 0.81).

### 3.2 Random effects

The prevalence of congenital anomaly‐related perinatal mortality in the null model (Model 0) varied between countries with a “baseline” between‐country variance (*τ*_00_) of 0.28 (95% CI 0.11, 0.70). In the model including only TOPFA (Model 1), the between‐country variation $\left(\tau_{00}^{*}\right)$ was 0.07 (95% CI 0.03, 0.18), meaning the prevalence of TOPFA accounted for 75.5% of the variation in congenital anomaly‐related perinatal mortality between countries (Table 2). In the model including only prenatal diagnosis only (Model 2), the between‐country variation $\left(\tau_{00}^{\psi }\right)$ was 0.17 (95% CI 0.07, 0.44), meaning the prevalence of prenatal diagnosis accounted for 37.7% of the between‐country variation in perinatal mortality. In the model adjusted for TOPFA and prenatal diagnosis of congenital anomaly prevalence (Model 3), the between‐country variation $\left(\tau_{00}^{*\psi }\right)$ was 0.05 (95% CI 0.02, 0.13), meaning 83.0% of the variation in congenital anomaly‐related perinatal mortality was accounted for.

**Table 2:** Fixed and random effects from models 1, 2, and 3 showing the associations between congenital anomaly‐related perinatal mortality prevalence with TOPFA and prenatal diagnosis of congenital anomaly prevalence

Indicator (per 1000 births)	Fixed effects: IRR (95% CI)	Random effects: between‐country variance (95% CI)
TOPFA (model 1)	0.79 (0.72, 0.86)	0.07 (0.03, 0.18)
Prenatal diagnosis (model 2)	0.88 (0.79, 0.97)	0.17 (0.07, 0.44)
TOPFA adjusted for prenatal diagnosis (model 3)	0.70 (0.61, 0.81)	0.05 (0.02, 0.13)

Abbreviations: IRR, incidence rate ratio; CI, confidence interval.

## 4 COMMENT

### 4.1 Principal findings

We have demonstrated a multilevel method for quantifying the degree of variation in one public health indicator that is attributable to another. Here, TOPFA prevalence was negatively associated with congenital anomaly‐related perinatal mortality prevalence and accounted for a large percentage of between‐country variation in perinatal mortality among babies with congenital anomalies. The prevalence of prenatal diagnosis of congenital anomalies was also negatively associated with congenital anomaly‐related perinatal mortality prevalence but accounted for a smaller proportion of between‐country variation in perinatal mortality prevalence among babies with congenital anomalies. These results show that it is important to understand variation in TOPFA rates when interpreting and comparing congenital anomaly‐related perinatal mortality rates between countries. However, even though perinatal mortality may be prevented via TOPFA, the adverse birth outcome and the physical or emotional burden to the parents still exists and so the two indicators must be considered in parallel.

### 4.2 Strengths of the study

This study has several strengths. Firstly, the EUROCAT public health indicators we have presented are well defined, therefore satisfying an important criteria of an effective indicator.^13^ EUROCAT uses these measures as quality indicators and due to the use of multiple sources of case ascertainment they are rigorously checked. Congenital anomaly‐related perinatal mortality is an important indicator that represents the burden of mortality and acts as a marker for quality of prenatal screening programmes, maternity care, and access/uptake of health services. Additionally, our results are arguably more robust because we excluded countries with potentially lower ascertainment of congenital anomaly‐related perinatal mortality, including only countries with infant mortality rates comparable or higher than those reported by the WHO.^11^ However, in applying this exclusion criteria, we cannot rule out the possibility that we excluded data from countries with genuinely low perinatal mortality rates.

Previous methods for analysing inter‐linked public health indicators include presenting the indicators graphically or estimating only IRRs.^1^, ^14^ Our proposed method allows the estimation of IRRs, but also provides a simple quantitative summary of the variation in one indicator that is attributable to one or more indicators. Although we have presented basic forms of the proposed technique, our approach is advantageous because it can be adapted to more complex scenarios. For example, we pooled the prevalence of the indicators by country in order to present the most simplistic form of the multilevel model. However, the prevalence also varied within country, for example prenatal diagnosis of congenital anomaly prevalence was 13.0 per 1000 in Basque Country but 6.9 per 1000 in Valencia Region of Spain. To account for this within country variation, we could have included an additional level within our multilevel model. A random slope could also be incorporated into the model to account for trends in public health indicators over time. It is also possible to test the hypothesis that there was a non‐linear association between indicators by incorporating fractional polynomials or splines. There is scope to include further variables such as demographic information on the study population, for example smoking rates, health service indicators or economic factors. It would also be possible to incorporate interactions between the public health indicators used as explanatory variables.

### 4.3 Limitations of the data

There are certain caveats and limitations in our study. While we can assess the relation between indicators and calculate the percentage of variation in one indicator that may be attributable to another, these represent empirical associations not causal ones. Contextual factors including those related to data issues and various possible connections between indicators should be considered in interpreting the statistical results from the proposed models. For example, we found a negative association between congenital anomaly‐related perinatal mortality prevalence and TOPFA prevalence; this association could be causal if severe cases of congenital anomaly are terminated more frequently and cannot contribute to perinatal mortality. Alternatively, increased termination rates may be acting as a proxy for improved access and/or uptake of health care or it could represent a more severe case mix of congenital anomalies, which would also influence perinatal mortality. The prevalence of TOPFA and congenital anomaly‐related perinatal mortality may vary between countries due to differences in the coding of late TOPFA, given that some countries may code these as stillbirths as opposed to TOPFA. As always, any causal interpretations of the estimates need to be made in light of substantive knowledge of the field, including its inherent uncertainties.

### 4.4 Interpretation

We found that increased prenatal diagnosis rates were associated with decreased risk of congenital anomaly‐related perinatal mortality. Countries with higher prenatal diagnosis of congenital anomaly prevalence had a higher prevalence of TOPFA, meaning the association may be mostly related to the impact of TOPFA. This is not surprising since prenatal diagnosis will not always result in a TOPFA but the vast majority of TOPFAs are likely to have been preceded by prenatal diagnosis. On the other hand, prenatal diagnosis of congenital anomaly can result in optimisation of pre‐ and postnatal care resulting in improved survival in babies with congenital anomalies. Prenatal diagnosis may also be a marker of improved health care access and services, which is thus associated with decreased congenital anomaly‐related perinatal mortality. Individual‐level data show conflicting evidence regarding the association between prenatal diagnosis and mortality. Several studies have reported no association between prenatal diagnosis and post‐operative survival in cases of congenital heart disease, the commonest type of congenital anomaly, although these are generally small studies that may be underpowered.^15^, ^16^, ^17^, ^18^ Hospital‐based studies have reported an increased risk of post‐operative mortality in prenatally diagnosed cases of congenital heart disease, most likely because more severe phenotypes are diagnosed prenatally.^19^, ^20^ However, in cardiovascular anomalies, the effect differs according to subtype.^21^

Additionally, we pooled the indicators over 4 years meaning changes in the prevalence of congenital anomaly‐related perinatal mortality, prenatal diagnosis of congenital anomaly and TOPFA were averaged over time. Although we studied a relatively short study period (2008‐12), this may have introduced some confounding of trends over time. With advances in prenatal diagnosis and medical and surgical interventions for congenital anomalies, congenital anomaly‐related perinatal mortality may have decreased and the associations with TOPFA and prenatal diagnosis may have altered in more recent years. Prevalence rates for the EUROCAT public health indicators discussed in this paper are updated annually on the EUROCAT website (https://eu-rd-platform.jrc.ec.europa.eu/eurocat/eurocat-data/key-public-health-indicators_en).

A Canadian study also reported a negative association between TOPFA (occurring 20‐23 weeks gestation) and infant deaths among cases of congenital anomaly after examining graphically their two indicators of interest.^22^ In the European population, there may be other differences that contribute to variation in congenital anomaly‐related perinatal mortality, for example differences in maternal age, parity, and multiple births are known to influence foetal and neonatal deaths, although with relatively small effect sizes.^23^ Factors such as maternal smoking, alcohol use, obesity, socio‐economic status, and diabetes are also known risk factors for perinatal mortality in general and vary by European country.^24^, ^25^, ^26^, ^27^, ^28^, ^29^, ^30^, ^31^ Additionally, we combined all types of congenital anomaly. Countries with a higher prevalence of more severe congenital anomalies but with respectively lower prevalence of prenatal diagnosis or TOPFA are likely to have had even higher perinatal mortality than that described by the model.

## 5 CONCLUSIONS

We have demonstrated a multilevel approach for quantifying the degree of variation in one public health indicator that may be attributable to another. In our example, we found a negative association between TOPFA rates and perinatal mortality among cases of congenital anomaly, which accounted for 75.5% of between‐country variation. Prenatal diagnosis with TOPFA and prenatal diagnosis modelled together accounted for 83% of between‐country variation in perinatal mortality.

## CONFLICTS OF INTEREST

None.
